# Noninvasive Orthogonal Polarization Spectral Imaging as Applied to Microvascular Studies in Mice

**DOI:** 10.1080/15438600490486859

**Published:** 2004

**Authors:** P. Nivoit, A. M. Chevrier, M. Lagarde, C. Renaudin, N. Wiernsperger

**Affiliations:** 1 Diabetic Microangiopathy Research Unit MERCK Santé–INSERM UMR 585 INSA-Lyon Louis Pasteur Bldg., 11 ave. Jean Capelle Villeurbanne Cedex 69621 France; 2 INSERM UMR 585 INSA-Lyon Villeurbanne France

## Abstract

In vivo observations of the mouse microcirculation can hardly be performed due to technical difficulties, limiting the knowledge that could be obtained from gene manipulated mice models. The aim of the present study was to check the applicability of a novel optical system, the orthogonal polarization spectral technology, to study the mouse microcirculation. In anaesthetized mice, the spinotrapezius muscle microcirculation was observed in situ. The diameter of precapillary arterioles was measured before and after a pharmacological or hormonal stimulation. High-contrast images of the muscle microcirculation were obtained and significant vasodilatation of arterioles was observed after topical applications of acetylcholine, sodium nitroprusside, and insulin. As compared to conventional techniques, orthogonal polarization spectral imaging makes it possible to assess and study microvascular beds in mice, which were inaccessible until now, allowing the use of gene manipulated mice to investigate, for example, the mechanisms involved in the development of diabetic microangiopathy.


*Experimental Diab. Res.*, 5:211–217, 2004
Copyright © Taylor and Francis Inc.
ISSN: 1543-8600 print / 1543-8619 online
DOI: 10.1080/15438600490486859

Taylor & Francis
Taylor & Francis Group

### Noninvasive Orthogonal Polarization Spectral Imaging as Applied to Microvascular Studies in Mice

**P. Nivoit,[1] A. M. Chevrier,[1] M. Lagarde,[2] C. Renaudin,[1] and N. Wiernsperger[1]**

[1]*Diabetic Microangiopathy Research Unit, MERCK Santé–INSERM UMR 585, INSA-Lyon, Villeurbanne Cedex, France*
[2]*INSERM UMR 585, INSA-Lyon, Villeurbanne, France*

**In vivo observations of the mouse microcirculation can hardly be performed due to technical difficulties, limiting the knowledge that could be obtained from gene manipulated mice models. The aim of the present study was to check the applicability of a novel optical system, the orthogonal polarization spectral technology, to study the mouse microcirculation. In anaesthetized mice, the spinotrapezius muscle microcirculation was observed in situ. The diameter of precapillary arterioles was measured before and after a pharmacological or hormonal stimulation. High-contrast images of the muscle microcirculation were obtained and significant vasodilatation of arterioles was observed after topical applications of acetylcholine, sodium nitroprusside, and insulin. As compared to conventional techniques, orthogonal polarization spectral imaging makes it possible to assess and study microvascular beds in mice, which were inaccessible until now, allowing the use of gene manipulated mice to investigate, for example, the mechanisms involved in the development of diabetic microangiopathy.**

**Keywords** Arteriole; Microcirculation; Mouse; Orthogonal Polarization Spectral Imaging; Skeletal Muscle

Microvascular pathologies are a key feature of many human disease states, including hypertension [1], coronary heart disease [2], and diabetes mellitus [3]. In diabetes, the so-called diabetic microangiopathy underlies retinopathy, nephropathy, and neuropathy. It is well recognized that these long-term complications of diabetes originate from malfunction of the microvasculature [4]. In industrialized countries, diabetic retinopathy remains the leading cause of blindness [5], diabetic nephropathy is the main cause of end-stage renal disease [6], and diabetic peripheral neuropathies are the major cause of lower limb amputation [7]. These data highlight the urgent need to clarify the processes involved in the pathogenesis of diabetic microcomplications in order to achieve efficient therapeutic strategies that can improve the microcirculatory function. However, the etiology of long-term diabetic microvascular complications is still poorly understood and this lack of knowledge can partly be explained by the technical difficulties linked to the observation of the microvasculature in situ [8].

Microcirculation is difficult to access because of its small dimensions and its location and therefore requires adapted techniques. In humans, conventional techniques available to observe the microcirculation are necessarily noninvasive, therefore limiting the investigations to vascular beds where vessels are visible and close to the surface such as skin, finger nail fold, or conjunctiva. However, this raises the question whether studies performed in these tissues are relevant for diabetes because (1) microvascular dysfunction during metabolic disorders occurs in many microvascular networks with more important physiological, functions, and (2) many tissues have their own physiological regulation. In animals, using invasive methods and video microscopy, deeper tissues can be observed, such as skeletal muscle (spinotrapezius, cremaster, extensor digitorum longus) or gastrointestinal tract (mesenter, colon). However, these microvascular preparations require extensive surgical procedures to exteriorize the organ, thus questioning their validity

Received 16 January 2004; accepted 26 March 2004.
Address correspondence to P. Nivoit, Diabetic Microangiopathy Research Unit, MERCK Santé–INSERM UMR 585, INSA-Lyon, Louis Pasteur Bldg., 11 ave. Jean Capelle, F-69621 Villeurbanne Cedex, France. E-mail: pierre.nivoit@insa-lyon.fr to represent the in vivo situation [9]. Classical microvascular preparations potentially exhibit also another drawback, because they are mostly impossible to adapt to animal species as small as mice. In vivo microcirculatory investigations are therefore currently limited to species such as rats or bigger animals. On the other hand, the field of metabolic disorders (insulin resistance, glucose intolerance, obesity, diabetes) has experienced an explosion of specific models over the past few years, which are essentially engineered by genetic manipulations in mice (protein overexpression or knockout). Investigating the microvascular modifications involved in the aggravation of metabolic dysfunction [8] or underlying diabetic vascular complications might increasingly require such models to unravel these functional connections.

Recently, a new technique for the imaging of microcirculation, the orthogonal polarization spectral (OPS) imaging [10], was developed. Compared to intravital microscopy, OPS imaging offered several advantages, making this device potentially advantageous to visualize the microcirculation in mice and to investigate quantitative parameters such as arteriolar diameter or functional capillary density changes [10]. Indeed, OPS imaging produces highly contrasted images of microvascular networks without the use of fluorescent dyes or elaborate and destructive tissue preparation for transillumination.

So, the aim of the present study was to check the applicability of the OPS imaging technology to study the mouse microcirculation when standard intravital microscopy (for example, transillumination) can hardly be performed. To this end, we adapted a previously described intact rat spinotrapezius muscle model [11] for the use in mice and validated its suitability for microvascular reactivity studies with vasoactive drugs and also its suitability for endocrine microvascular studies.

### MATERIALS AND METHODS

### Animals

Male C57Bl6/J mice (Charles River, l'Arbresle, France) weighing 26 ± 1 g were used for the experiments. The animals were kept under standard conditions (light 07.00 to 19.00 hours; temperature 22°C ± 1°C; humidity 50% ± 10%), and fed rodent diet and water ad libitum.

### Spinotrapezius Muscle Preparation

All surgical and experimental procedures were performed under constant flow of isoflurane anaesthesia (2.0% to 2.5%) in oxygen–nitrous oxide gas mixture (40% to 60%). Throughout the whole experiment, body temperature was kept at 37°C ± 1°C by placing mouse in supine position on a complete homeothermic blanket system (Harvard Apparatus, Les Ulis, France). Polyethylene catheters (PE-10; Intramedic, FLD, Chilly Mazarin, France) were inserted into the carotid artery for blood pressure measurement. The rat spinotrapezius muscle preparation [11] was adapted and simplified for use in mouse: a longitudinal slit was performed in the skin along the spine from the cervical to the midlumbar region and the connective tissue that covers the muscle was carefully removed using iridectomy scissors. During the preparation and experimental observations, the skeletal tissue was continuously superfused with a bicarbonate/HEPES-buffered saline. The temperature of the solution was kept at 36°C and the pH was set at 7.4 by bubbling the solution with 5% $CO_2$ in 95% $N_2$. Superfusion flow rate was maintained at 4 to 5 mL/min (Figure 1).

### Orthogonal Polarization Spectral Imaging Technology

The OPS imaging technology, described in detail by Groner and colleagues [10], allowed visualizing the microcirculation. Briefly, the technique eliminates directly reflected green polarized light from an organ surface using an orthogonal placed analyzer, which results in clear intravital images of red blood cells flowing through the microcirculation. For our study, we used the OPS imaging Cytoscan Video Microscope (Cytometric, Philadelphia, USA) with the 10× probe positioned on the top of the muscle that allowed the observation of microcirculatory fields of 0.47 × 0.63 mm. Images were captured and recorded on S-VHS videotape and analyzed off-line.

### Image Analysis

Arteriolar diameters were determined by measurements of the red blood column diameters, the median value of at least 5 consecutive diameter measurements being considered as the real diameter. This was performed by a playback analysis of the video record using an image processing system. The image was coded as 768 by 572 pixels, with 256 gray levels. The isotropic orientation of the vessel was used to increase the signal/noise ratio, by integration of the segments perpendicular to the operator's defined line drawn on the vessel. The diameters were computed at 50% of the gray level amplitude of the integrated signal.

### Blood Pressure Measurements

The arterial blood pressure signal, transmitted to a blood pressure analyser (AH 60-3003, Harvard Apparatus, Les Ulis, France), was digitized at 1000 samples per second. Each second, mean blood pressure and heart rate were computed, displayed, and stored using dedicated software IOX (EMKA Technology, Paris, France).

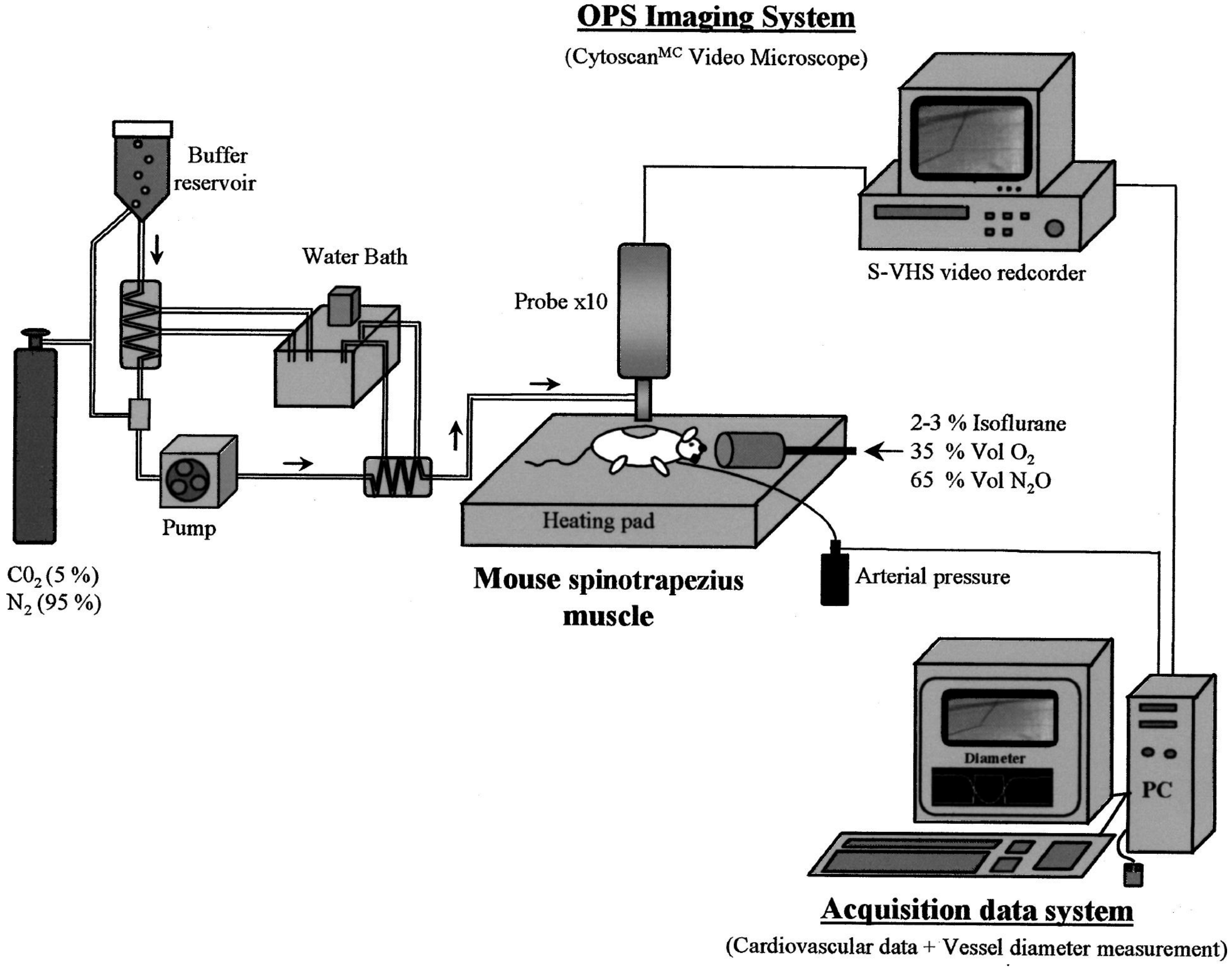

**FIGURE 1**
Schematic experimental setup.

### Experimental Protocols

Mice were allowed a 45-minute stabilization period before the experimental protocols were begun. Four regions of interest within the spinotrapezius muscle that contained one or more terminal precapillary arterioles (smaller than 20 $\mu$m) were selected. For measurement, the 4 microcirculatory fields were recorded for ~60 seconds each; the value of arteriolar diameter at this time will be considered as basal diameter.

In a first series of experiments, we examined responses of mouse spinotrapezius arterioles to the endothelium-dependent vasodilator acetylcholine (Ach; 0.1 $\mu$ and 1.0 $\mu$; n = 4). We also examined dilation of arterioles to the endothelium-independent nitric oxide donor sodium nitroprusside (SNP; 0.1 $\mu$M and 1.0 $\mu$M; n = 4). After baseline measurements, superfusion with bicarbonate buffer was interrupted and acetylcholine or sodium nitroprusside prepared in the same medium was topically applied. Arteriolar diameters were measured after 3 minutes of the topical application. In pilot experiments, maximal vasodilatory effects were obtained at the concentration of 1 $\mu$M. Thus, the concentration of 0.1 $\mu$M and 1.0 $\mu$M were selected in each case.

In a second series of experiments, mice were randomized into either the control group (n = 8) or insulin group (n = 8). In mice of the control group, physiological buffer solution was superfused throughout the entire experiment. In mice of the insulin group, 200 $\mu$IU/mL insulin (Actrapid, Novo Nordisk Pharmaceutique S.A., Boulogne, France) diluted in physiological buffer, was superfused for 45 minutes. Video recoding was done at 15, 30, and 45 minutes after the start of superfusion for later off-line analysis.

### Statistical Analysis

Results are expressed as mean ± SEM. Between-group comparisons of mice metabolic parameters were performed using the Student-Newman-Keuls'post hoc pairwise test. The data for arteriolar diameters were analyzed using 2-way analysis of variance (ANOVA) to identify time and drug effects. When a significant value was demonstrated, 1-way ANOVA was used, followed by the Tukey's post hoc analysis, to analyze the time effect in each experimental group and drug effect for each series of experiment. A $P$ value of .05 or less was considered statistically significant. Data were analyzed using the statistical software SPSS (SPSS France, Paris, France).

### RESULTS

### OPS Imaging

High-contrast images of terminal precapillary arterioles of the mouse spinotrapezius muscle were obtained by the OPS imaging system (Figure 2), allowing the measurement of vascular diameter.

### Baseline Data of the Mouse Spinotrapezius Model

Basal physiologic parameters of C57Bl6/J mice were similar in all groups: mean arterial blood pressure was 90 ± 5 mm Hg, heart rate was 545 ± 13 bpm, and precapillary arteriolar diameter established at 10.4 ± 0.5 $\mu$m. No significant changes of mean blood pressure and heart rate were observed during the experiments.

### Effect of Vasoactive Drugs on Arteriolar Diameters

Figure 3 summarizes the effect of topical application of acetylcholine and sodium nitroprusside on the precapillary arteriolar diameter. Acetylcholine and sodium nitroprusside produced dose-related dilation of arterioles. Topical acetylcholine (0.1 $\mu$M and 1.0 $\mu$M) caused ~20% and ~35% increases over the initial arteriolar diameters, respectively, whereas topical sodium nitroprusside (0.1 $\mu$M and 1.0 $\mu$M) caused ~25% and ~45% increases over the initial arteriolar diameters, respectively.

### Effect of Insulin on Precapillary Arteriolar Diameters

Arteriolar diameters in the spinotrapezius muscle remained stable over time in animals injected with isotonic saline. On the contrary, a pronounced vasodilation was induced by insulin (Figure 4). Precapillary arteriolar diameters were significantly increased at time 15 minutes (8.5%) and the effect persisted until the end of the experiment to reach the value of 12.3% at time 45 minutes.

### DISCUSSION

The basic rationale for this study emerged from the facts that the pathophysiology of diabetic microangiopathy is still poorly understood. To fully understand the functional modifications

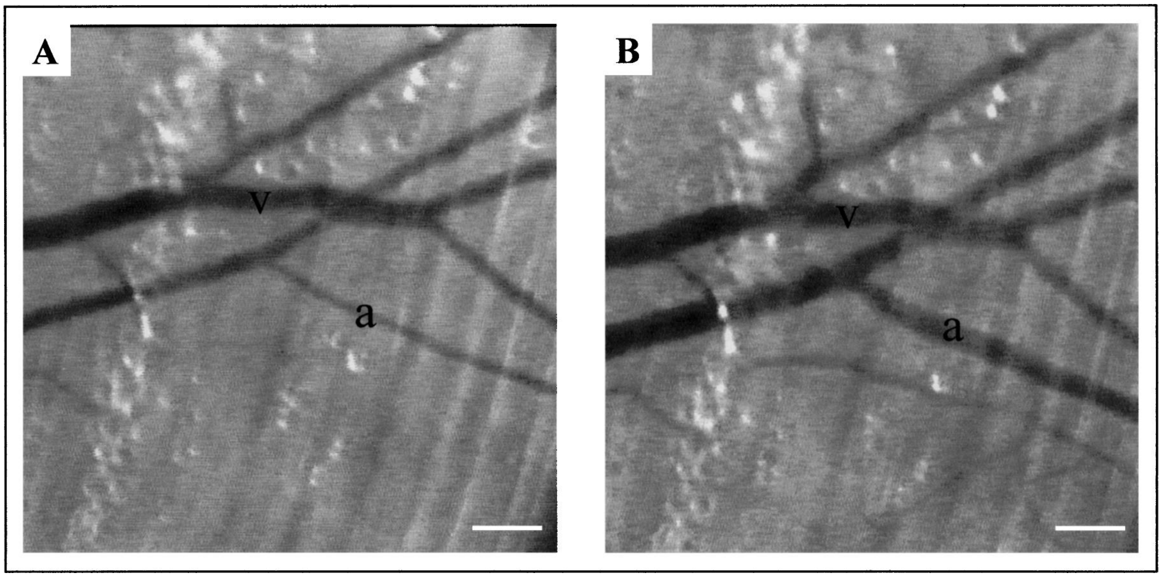

**FIGURE 2**
Representative examples of high-contrast images obtained in vivo by OPS imaging (Cytoscan Video Microscope) of the mouse spinotrapezius muscle arterioles (a) and venules (b) before ($A$) and after ($B$) topical application of acetylcholine (10 $\mu$M). Scale bar represent 100 $\mu$m.

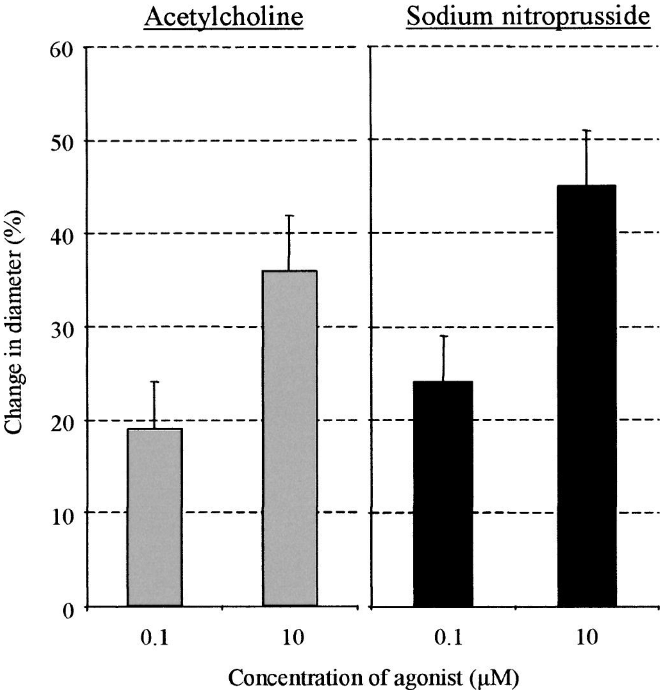

**FIGURE 3**
Response of the mouse spinotrapezius muscles precapillary arterioles to topical application of acetylcholine (*gray bars*) and sodium nitroprusside (*black bars*). Data are means ± SEM.

involved in these long-term vascular complications, direct microcirculatory observations have to be made in animal models, the prerequisite for development of therapeutic strategies in humans. With the introduction of genetically modified mice

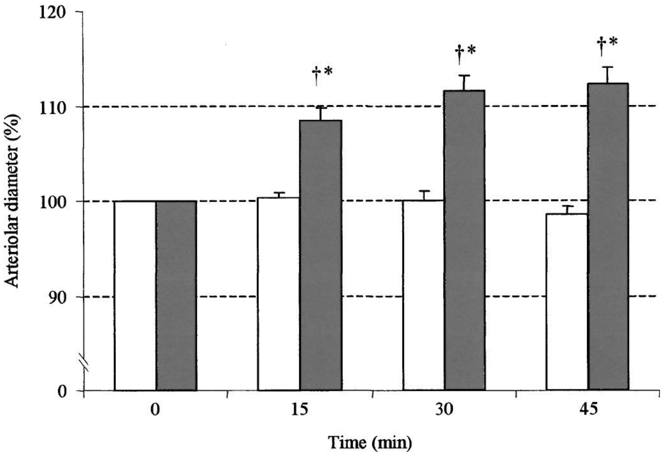

**FIGURE 4**
Effects of insulin on the skeletal muscle precapillary arteriolar diameters in mice. Insulin (*gray bars*) was superfused at a dose of 200 $\mu$IU/mL over 45 minutes. In control mice (*white bars*), physiological buffer was superfused throughout the whole experiment. Data are means ± SEM. $^{*}P < .05$ versus saline, $^{\dagger}P < 0.05$ versus time 0.

in the life sciences, murine models will provide invaluable research tools for understanding both the biological pathways and the etiology of diabetic microangiopathy. Until now, microvascular preparations used in rats or hamsters for intravital microscopy were, however, difficult to adapt in mice due to the extensive surgical procedures needed to exteriorize the observed tissue for transillumination. These problems may now become resolved by the availability of a new method for the study of microcirculation: the orthogonal polarization spectral imaging or OPS [10].

OPS imaging is a novel optical system that produces highly contrasted images of microvascular networks. The technique relies on the hemoglobin absorption of green polarized light to enhance the contrast of the red blood cells in the microvessels. Unlike classical intravital microscopy, there is no more need to transilluminate the tissue or to use fluorescent (possibly toxic) dyes for contrast enhancement. Microcirculation can therefore be observed noninvasively through mucus membrane and on the surface of various solid organs. In recent validation studies, it was shown that OPS imaging was as efficient as standard intravital microscopy in quantifying microvascular characteristics such as vessels diameter, red blood cells velocity, and functional capillary density [10, 12–16]. Observations were made in physiological conditions but also in pathophysiological conditions, including inflammation [13], ischemia [16], and hemodilution [15].

Few studies report the use of mice in experimental settings [13, 14, 17] and only one was aiming at validating the use of OPS imaging against standard intravital fluorescence microscopy in a microcirculatory preparation of mouse cremaster muscle [17]. In the current study, we choose to investigate the mouse microcirculation with OPS imaging in the spinotrapezius muscle for several reasons. Firstly, the spinotrapezius muscle was used historically as the first model for the intravital observation in rats [18]. Secondly, the rat spinotrapezius muscle preparation performed according to the method described by Gray [11] is currently in use in our laboratory [19, 20]. Thirdly, histochemical characteristics and contractile properties of the spinotrapezius muscle of rats and mice are very similar [21]. Because standard intravital videomicroscopy cannot be carried out in the mouse due to insufficient length of muscle to be exteriorized, it explains why mouse spinotrapezius muscle, in contrast to cremaster, has not yet been adapted to mice [22]. Finally, skeletal muscle is one of the major insulin-sensitive tissues [23] and insulin has been implicated in the diabetic microvascular hemodynamic defects, which could contribute to the pathogenesis of diabetic microangiopathy [8, 24].

Until now, intravital microscopy enabled observation in 2 mouse skeletal muscle models: the cremaster [22, 25] and the extensor digitorum longus models [26]. In the mouse spinotrapezius model, utilization of OPS imaging now avoids one major drawback encountered with these models, namely complex and time-consuming surgical procedures, which furthermore increase the risk of damaging the preparation. Indeed in the cremaster muscle preparation, the muscle needs to be completely separated from the testicle by cutting the small linking pedicle. In extensor digitorum longus preparation, the tibialis anterior muscle tendon needs to be transected to put backwards the muscle that covers the extensor digitorum longus muscle. In contrast, preparation of spinotrapezius appears very simple, just requiring an incision of the neck skin in order to expose the muscle, followed by careful removal of the connective tissue. Application of the OPS imaging probe to the surface of the muscle at a working distance of 2 mm eliminates any mechanical stresses, which could alter microvascular flow [27]. In addition, the field depth of the optical system, combined with the thinness of the muscle, allows visualizing the microcirculation via the dorsal strip of the muscle in a situation that truly represents the in vivo situation [9]. As demonstrated in Figure 2, high-contrast images from the microvascular network of spinotrapezius were obtained. This was the necessary condition to measure arteriolar diameter off-line. Due to the simplicity of accessing skeletal muscle (skin incision), the OPS may also allow to move to more chronic (repetitive) investigations, which are of primary importance for studying the evolution in metabolic disorders.

In a first approach, we validated our model by monitoring arteriolar diameter throughout the whole experiment during superfusion with bicarbonate buffer. Our data show that precapillary arteriolar diameter remains constant, therefore demonstrating the stability of the microvascular preparation. Next we verified the suitability of our model to probe endothelial function because endothelial dysfunction is known to play a key role in the development of diabetic microvascular complications [28, 29]. We therefore investigated the acetylcholine-induced vasodilation, which has become a standard test used to assess the endothelial function and has been generally found to be impaired in diabetes, at both the macrovascular and microvascular levels [30]. However, impaired endothelium-dependent vasodilation is frequently observed in the presence of preserved responses to endothelium-independent vasodilation. This prompted us to test the endothelium-independent vasodilation induced by sodium nitroprusside, a NO donor that stimulates the relaxation of the smooth muscle cells. As demonstrated in Figure 3, topical applications of acetylcholine or sodium nitroprusside both induced precapillary arteriolar vasodilation in a dose-dependent manner, validating the suitability of our model for microvascular reactivity studies. Finally, the suitability of this technique was also tested for endocrine vascular studies. Indeed, it is well established that besides its metabolic action, insulin can affect blood flow in skeletal muscle [24, 31]. Insulin alone induces vasodilation in large arteries and resistance vessels and we recently showed that this effect also occurs in precapillary arterioles of the rat spinotrapezius muscle [19]. We therefore investigated the effect of a postprandial insulin concentration [32] on precapillary arteriolar tone. As expected, the results of our study showed that superfusion of insulin reduced significantly precapillary arteriolar tone in mouse spinotrapezius muscle. Our results are in good agreement with those of Wehrens and colleagues in extensor digitorum longus muscle preparation where the same dose of insulin induced a similar vasodilation around 10% [26]. In addition, precapillary arteriolar vasodilation was also observed when insulin was injected subcutaneously (data not shown). By modifying the precapillary arteriolar dynamics, insulin may increase capillary recruitment and therefore facilitate the delivery of glucose and hormone to muscle cells, leading to increased glucose uptake [8, 24, 31]. Impairment of this process could therefore lead to—or aggravate—insulin resistance and subsequently be involved early in the pathogenesis of diabetes as well as its microvascular complications [8, 24]. However, this concept is still debated and we hypothesize that, using gene manipulated mice combined with OPS imaging in experimental settings, further investigations would help to unravel some yet unanswered questions about such linkings.

In summary, we examined the transfer and applicability of the OPS imaging technology to investigate the mouse microcirculation. As compared to conventional technique/models, OPS makes it possible to greatly reduce the level of surgery to access the microvascular bed of skeletal muscles, a tissue of primary importance in the pathophysiology of metabolic disorders. This technology will now provide a unique opportunity by allowing investigation of the pathophysiology of diabetic microangiopathy as an extension to gene manipulated mice.

### REFERENCES

[1] Vicaut, E. (2003) Hypertension and the microcirculation. *Arch. Mal. Coeur. Vaiss.,* **96,** 893–903.

[2] Hasdai, D., Gibbons, R. J., Holmes, D. R., Jr., Higano, S. T., and Lerman, A. (1997) Coronary endothelial dysfunction in humans is associated with myocardial perfusion defects. *Circulation,* **96,** 3390–3395.

[3] Hammes, H. P. (2003) Pathophysiological mechanisms of diabetic angiopathy. *J. Diabetes Complications,* **17**(Suppl 2), 16–19.

[4] Tooke, J. E. (1996) Microvasculature in diabetes. *Cardiovasc. Res.,* **32,** 764–771.

[5] Porta, M., and Bandello, F. (2002) Diabetic retinopathy. A clinical update. *Diabetologia,* **45,** 1617–1634.

[6] Bommer, J. (2002) Prevalence and socio-economic aspects of chronic kidney disease. *Nephrol. Dial. Transplant.,* **17**(Suppl 11), 8–12.

[7] Thomas, P. K. (1999) Diabetic peripheral neuropathies: Their cost to patient and society and the value of knowledge of risk factors for development of interventions. *Eur. Neurol.,* **41**(Suppl 1), 35–43.

[8] Wiernsperger, N. (2000) Defects in microvascular haemodynamics during prediabetes: Contributor or epiphenomenon? *Diabetologia,* 43, 1439–1448.

[9] Nolte, D., Menger, M. D., and Messmer, K. (1995) Microcirculatory models of ischaemia-reperfusion in skin and striated muscle. *Int. J. Microcirc. Clin. Exp.,* **15,** 9–16.

[10] Groner, W., Winkelman, J. W., Harris, A. G., Ince, C., Bouma, G. J., Messmer, K., and Nadeau, R. G. (1999) Orthogonal polarization spectral imaging: A new method for study of the microcirculation. *Nat. Med.,* **5,** 1209–1212.

[11] Gray, S. D. (1973) Rat spinotrapezius muscle preparation for microscopic observation of the terminal vascular bed. *Microvasc. Res.,* **5,** 395–400.

[12] Harris, A. G., Sinitsina, I., and Messmer, K. (2000) The Cytoscan Model E-II, a new reflectance microscope for intravital microscopy: Comparison with the standard fluorescence method. *J. Vasc. Res.,* **37,** 469–476.

[13] Biberthaler, P., Langer, S., Luchting, B., Khandoga, A., and Messmer, K. (2001) In vivo assessment of colon microcirculation: Comparison of the new OPS imaging technique with intravital microscopy. *Eur. J. Med. Res.,* **17,** 525–534.

[14] Langer, S., Born, F., Hatz, R., Biberthaler, P., and Messmer, K. (2002) Orthogonal polarization spectral imaging versus intravital fluorescent microscopy for microvascular studies in wounds. *Ann. Plast. Surg.,* **48,** 646–653.

[15] Harris, A. G., Sinitsina, I., and Messmer, K. (2002) Validation of OPS imaging for microvascular measurements during isovolumic hemodilution and low hematocrits. *Am. J. Physiol. Heart. Circ. Physiol.,* **282,** 1502–1509.

[16] Langer, S., Von Dobschuetz, E., Harris, A. G., Krombach, F., and Messmer, K. (2000) Validation of the orthogonal polarization spectral imaging technique on solid organs. In: *Progress in Applied Microcirculation (Vol. 24), Orthogonal Polarization Spectral Imaging, A New Tool for the Observation and Measurements of the Human Microcirculation*, Edited by Messmer, K., pp. 32–46. Basel, Karger.

[17] Laemmel, E., Tadayoni, R., Sinitsina, I., Boczkowski, J., and Vicaut, E. (2000) Using Orthogonal spectral imaging for the experimental study of microcirculation: Comparisons with intravital microscopy. In: *Progress in Applied Microcirculation (Vol. 24), Orthogonal Polarization Spectral Imaging, A New Tool for the Observation and Measurements of the Human Microcirculation,* Edited by Messmer, K., pp. 50–60. Basel, Karger.

[18] Zweifach, B. W., and Metz, D. B. (1955) Selective distribution of blood through the terminal vascular bed of mesenteric structures and skeletal muscle. *Angiology,* **6,** 282–290.

[19] Renaudin, C., Michoud, E., Rapin, J. R., Lagarde, M., and Wiernsperger, N. (1998) Hyperglycaemia modifies the reaction of microvessels to insulin in rat skeletal muscle. *Diabetologia,* **41,** 26–33.

[20] Renaudin, C., Michoud, E., Lagarde, M., and Wiernsperger, N. (1999) Impaired microvascular responses to acute hyperglycemia in type I diabetic rats. *J. Diabetes Complications,* **13,** 39–44.

[21] Taylor, K., and Calvey, T. N. (1977) Histochemical characteristics and contractile properties of the spinotrapezius muscle in the rat and the mouse. *J. Anat.,* **123,** 67–76.

[22] Vicaut, E., and Stucker, O. (1990) An intact cremaster muscle preparation for studying the microcirculation by in vivo microscopy. *Microvasc. Res.,* **39,** 120–122.

[23] Baron, A. D., Brechtel, G., Wallace, P., and Edelman, S. V. (1988) Rates and tissue sites of non-insulin- and insulin-mediated glucose uptake in humans. *Am. J. Physiol.,* **255,** 769–774.

[24] Rattigan, S., Barrett, E. J., and Clark, M. G. (2003) Insulin-mediated capillary recruitment in skeletal muscle: Is this a mediator of insulin action on glucose metabolism? *Curr. Diab. Rep.,* **3,** 195–200.

[25] Mempel, T. R., Moser, C., Hutter, J., Kuebler, W. M., and Krombach, F. (2003) Visualization of leukocyte transendothelial and interstitial migration using reflected light oblique transillumination in intravital video microscopy. *J. Vasc. Res.,* **40,** 435–441.

[26] Wehrens, X. H., Van Breda, E., Van Velzen, J. S., Oude Egbrink, M. G., and Slaaf, D. W. (2000) Use of an intact mouse skeletal muscle preparation for endocrine vascular studies: Evaluation of the model. *Horm. Metab. Res.,* **32,** 378–380.

[27] Tyml, K., and Budreau, C. H. (1991) A new preparation of rat extensor digitorum longus muscle for intravital investigation of the microcirculation. *Int. J. Microcirc. Clin. Exp.,* **10,** 335–343.

[28] Tooke, J. E. (2000) Possible pathophysiological mechanisms for diabetic angiopathy in type 2 diabetes. *J. Diabetes Complications,* **14,** 197–200.

[29] Laight, D. W., Carrier, M. J., and Anggard, E. E. (1999) Endothelial cell dysfunction and the pathogenesis of diabetic macroangiopathy. *Diabetes. Metab. Res. Rev.,* **15,** 274–282.

[30] De Vriese, A. S., Verbeuren, T. J., Van de Voorde, J., Lameire, N. H., and Vanhoutte, P. M. (2000) Endothelial dysfunction in diabetes. *Br. J. Pharmacol.,* **130,** 963–974.

[31] Clark, M. G., Wallis, M. G., Barrett, E. J., Vincent, M. A., Richards, S. M., Clerk, L. H., and Rattigan, S. (2003) Blood flow and muscle metabolism: A focus on insulin action. *Am. J. Physiol. Endocrinol. Metab.,* **284,** 241–258.

[32] McKay, M. K., and Hester, R. L. (1996) Role of nitric oxide, adenosine, and ATP-sensitive potassium channels in insulin-induced vasodilation. *Hypertension,* **28,** 202–208.

